# Effects of cerebellar transcranial direct current stimulation on rehabilitation of upper limb motor function after stroke

**DOI:** 10.3389/fneur.2023.1044333

**Published:** 2023-03-16

**Authors:** Qiuwen Gong, Rubing Yan, Han Chen, Xia Duan, Xiaoyu Wu, Xin Zhang, Yi Zhou, Zhou Feng, Ya Chen, Jianbo Liu, Peng Xu, Jing Qiu, Hongliang Liu, Jingming Hou

**Affiliations:** ^1^Department of Rehabilitation, Southwest Hospital, Third Military Medical University (Army Medical University), Chongqing, China; ^2^School of Life Science and Technology, University of Electronic Science and Technology of China, Chengdu, China; ^3^School of Mechanical and Electrical Engineering, University of Electronic Science and Technology of China, Chengdu, China

**Keywords:** transcranial direct current stimulation, stroke, cerebellum, rehabilitation, upper limb motor function

## Abstract

**Background:**

The cerebellum is involved in the control and coordination of movements but it remains unclear whether stimulation of the cerebellum could improve the recovery of upper limb motor function. Therefore, this study aimed to explore whether cerebellar transcranial direct current stimulation (tDCS) therapy could promote the recovery of upper limb motor function in patients who suffered a stroke.

**Methods:**

In this randomized, double-blind, and sham-controlled prospective study, 77 stroke patients were recruited and randomly assigned to the tDCS group (*n* = 39) or the control group (*n* = 38). The patients received anodal (2 mA, 20 min) or sham tDCS therapy for 4 weeks. The primary outcome was the change in the Fugl-Meyer Assessment-Upper Extremity (FMA-UE) score from baseline to the first day after 4 weeks of treatment (T1) and 60 days after 4 weeks of treatment (T2). The secondary outcomes were the FMA-UE response rates assessed at T1 and T2. Adverse events (AEs) related to the tDCS treatment were also recorded.

**Results:**

At T1, the mean FMA-UE score increased by 10.7 points [standard error of the mean (SEM) = 1.4] in the tDCS group and by 5.8 points (SEM = 1.3) in the control group (difference between the two groups was 4.9 points, *P* = 0.013). At T2, the mean FMA-UE score increased by 18.9 points (SEM = 2.1) in the tDCS group and by 12.7 points (SEM = 2.1) in the control group (the difference between the two groups was 6.2 points, *P* = 0.043). At T1, 26 (70.3%) patients in the tDCS group had a clinically meaningful response to the FMA-UE score compared to 12 (34.3%) patients in the control group (the difference between the two groups was 36.0%, *P* =0.002). At T2, 33 (89.2%) patients in the tDCS group had a clinically meaningful response to the FMA-UE score compared with 19 (54.3%) patients in the control group (the difference between the two groups was 34.9%, *P* = 0.001). There was no statistically significant difference in the incidence of adverse events between the two groups. In the subgroup analysis of different hemiplegic sides, the rehabilitation effect of patients with right hemiplegia was better than that of patients with left hemiplegia (*P* < 0.05); in the age subgroup analysis, different age groups of patients did not show a significant difference in the rehabilitation effect (*P* > 0.05).

**Conclusion:**

Cerebellar tDCS can be used as an effective and safe treatment to promote recovery of upper limb motor function in stroke patients.

**Trial registration:**

ChiCTR.org.cn, identifier: ChiCTR2200061838.

## Introduction

Upper limb motor dysfunction is one of the most common functional impairments in patients who suffered a stroke. A previous study has shown that approximately 80% of patients in the acute phase of a stroke had upper limb motor impairment, and only approximately one-third of them achieved full functional recovery (1). Upper limb motor dysfunction seriously affected the activities of daily living and social participation of patients, and it was considered one of the most distressing long-term consequences of a stroke (2). Therefore, intervention in improving the upper limb motor function has been identified as one of the top 10 research priorities by stroke patients, caregivers, and clinicians (3).

Recently, several rehabilitation interventions have been used to improve upper limb motor function after a stroke, including movement therapy, physical agent modalities, and robot-assisted training (4). Although these treatments improved to some extent the upper limb function of patients who suffered a stroke, 50% of patients still have upper limb motor impairments even 4 years after the stroke (5). Therefore, it is essential to explore new rehabilitation strategies to effectively improve the upper limb function of patients who suffered a stroke.

Transcranial direct current stimulation (tDCS) is a non-invasive neuromodulation technique that uses constant microcurrent to regulate the activity of cerebral cortex neurons (6). It might be an effective neuromodulation technique for neurological rehabilitation (7). In recent years, studies have reported that tDCS improved the motor function of stroke patients, including upper limb function (8). However, these studies mainly concentrated on stimulating the primary motor cortex (M1). A recent meta-analysis study showed that using M1 as the stimulation target, tDCS only leads to significant improvements in upper limb function in patients with chronic disease but not in patients with acute or subacute strokes (9). Therefore, for these patients, it is necessary to find other stimulation targets to promote the recovery of upper limb function.

The cerebellum is one of the important motor regulation centers of the human body and is involved in maintaining body balance, regulating muscle tension, and coordinating voluntary movement. Our previous animal experiment showed that the cerebellum could play an essential role in motor learning (10). Other studies demonstrated the crucial role of the cerebellum in motor learning (11, 12). Recent evidence indicated that cerebellar stimulation could improve the balance and gait function in patients with Parkinson's disease (PD) and older adults (13, 14). According to the aforementioned evidence, in the present study, it was hypothesized that cerebellar tDCS treatment could be a new and effective intervention for the rehabilitation of upper limb function in patients with stroke. Given that the efficacy of cerebellar tDCS on stroke remains unknown, a randomized controlled trial (RCT) was conducted to first explore whether the application of tDCS in the cerebellum could promote the recovery of upper limb motor function in patients with stroke.

## Methods

### Participants

From 1 November 2018 to 30 November 2020, stroke patients from the Southwest Hospital of Army Medical University (China) were enrolled continuously. Inclusion criteria were as follows: (1) within 2 weeks to 6 months after a first-time unilateral ischemic or hemorrhagic stroke, where the stroke was diagnosed by computed tomography (CT) or magnetic resonance imaging (MRI); (2) patients aged 20–80 years; (3) unilateral upper limb motor dysfunction; and (4) right-handedness. Exclusion criteria were as follows: (1) cases involving bilateral hemispheres, brainstem, or cerebellar stroke; (2) severe upper limb spasticity (modified Ashworth scale grade ≥3); (3) the presence of pacemakers or intracranial metal implants; (4) cognitive impairment, severe aphasia, or psychiatric diagnoses, etc.; (5) any medical conditions precluding participation in medical examinations, such as infections; or (6) a history of epilepsy, brain tumor, and cranial surgery. All patients provided written informed consent. The study protocol was performed in accordance with the Declaration of Helsinki, and it was approved by the ethics committee of the local institution (Approval No. KY2021036). This trial was also registered at the ChiCTR.org.cn website (identifier: ChiCTR 2200061838).

### Study design

**Figure 1:**
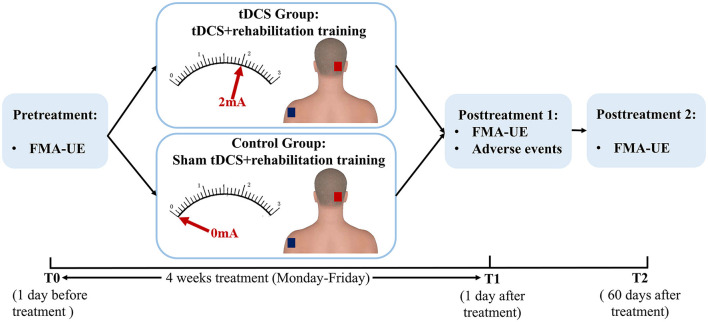
Schematic diagram of the experimental design. tDCS, transcranial direct current stimulation; FMA-UE, Fugl-Meyer Assessment-Upper Extremity.

This was a randomized, double-blind, and sham-controlled prospective study. The patients were randomly assigned to the tDCS group (*n* = 39, standard rehabilitation training combined with cerebellar tDCS therapy) or the control group (*n* = 38, standard rehabilitation training combined with cerebellar sham tDCS therapy) (Figure 1). Randomization of all patients was performed by generating a random allocation sequence using an online program (QuickCalcs: http://www.graphpad.com/quickcalcs/index.cfm). Throughout the study, a trained and experienced therapist with more than 5 years of work experience was responsible for the application of tDCS treatment. The outcome measures were evaluated by an attending physician who was trained before the study. Due to the nature of the intervention, the tDCS operator was aware of the patient grouping. The evaluator only contacted patients during the assessment and was blinded to the patient grouping. All rehabilitation therapists and stroke patients were blinded to the sham or active application of the tDCS.

### Intervention

Once patients were randomly assigned to one of the two groups, they received the same rehabilitation training in the neurorehabilitation unit. Patients received standard physical therapy (e.g., the Bobath approach, proprioceptive neuromuscular facilitation techniques, motor learning program, and constraint-induced movement therapy) that was based on their motor function. Specifically, standard therapy included the following items: (1) bed positioning and range of motion exercise, (2) bridge pose, (3) sitting and balance training, (4) standing and weight shifts, (5) sit-to-stand training, (6) standing balance exercise, and (7) walking training. The aforementioned rehabilitation treatments lasted for 2 h each time, 5 days a week (Monday to Friday), for 4 weeks.

Before the conventional rehabilitation training, patients received either cerebellar anodal tDCS or sham tDCS treatment. The tDCS stimulation was delivered by a portable stimulator (Zhejiang University R&D, T003) through a pair of 5 × 7 cm^2^ electrodes filled with a conducting gel. The anodal electrode was placed on the right cerebellum (3-cm right lateral to the inion), and the cathodal electrode was placed on the contralateral shoulder. The electrodes were fixed using rubber straps. Stimulation protocol for the tDCS group was summarized as follows: tDCS lasted 20 min at 2 mA, with ramp up and ramp down of 30 s. Participants in the control group received a 0.5 mA ramp up of 30 s, followed by a ramp down of 30 s, 19 min of 0 mA current ending with a 0.5 mA ramp up of 30 s, and a ramp down of 30 s. This protocol showed to be efficient for blinding patients (15). To avoid the presence of fatigue, tDCS was performed before the conventional rehabilitation training. Patients in both groups received stimulation once a day, 5 days a week, for 4 weeks.

### Outcome measures

The upper limb motor function of patients was assessed using the Fugl-Meyer Assessment-Upper Extremity (FMA-UE), a widely used stroke-specific, performance-based motor impairment index (16). This index is comprised of 33 items, with a score of 0–2 for each item. Total scores range from 0 to 66, and higher scores indicate lower levels of damage. The upper limb motor function of patients was assessed 1 day before tDCS treatment (T0), on the first day after 4 weeks of tDCS treatment (T1), and 60 days after the end of tDCS treatment (T2). The same evaluator performed assessments at baseline and follow-up.

The primary outcome was the change in the FMA-UE score compared with the baseline at T1 and T2. Secondary outcomes were as follows: (1) Clinically meaningful response rates for FMA-UE scores at T1 and T2. Referring to the previous studies on the upper limb motor function of patients with stroke (17), a clinically meaningful response was defined as an increase in the FMA-UE score of 6 points or more. (2) Patient-reported adverse events (AEs) related to tDCS treatment that occurred during the 4-week treatment period, such as tingling or itching under the electrodes, headache, fatigue, nausea, and insomnia during or after the intervention.

### Statistical analysis

The minimum sample size was estimated by G Power 3.1 statistical software (18). The parameters were summarized as follows: test family using “*t*-tests,” statistical test using “Means: Difference between two independent means (two groups),” and type of power analysis using “A priori: Compute required sample size.” To achieve a statistical power of 85% with statistical significance at *P* < 0.05 (two-sided test) and an effect size of *d* = 0.65, a minimum sample size of 70 patients was required. Considering the dropout rate of approximately 10% during the trial, the sample size of this study was expanded to 38 patients in each group.

Data were statistically analyzed using SPSS 21.0 software (IBM, Armonk, NY, USA). The Shapiro–Wilk test was used to examine the normality of the data. The results were presented as mean ± standard error of the mean (SEM) for continuous data and as number (percentage) for categorical data. Baseline group differences were compared using a *t*-test or chi-squared test. Repeated measures analysis of variance (rmANOVA) was applied to all outcome measures. The “time” point was used as the within-patient factor and “treatment” as the between-patient factor. *Post-hoc* analysis was performed using the Bonferroni correction for further multiple comparisons. *P* < 0.05 was considered statistically significant.

## Results

**Figure 2:**
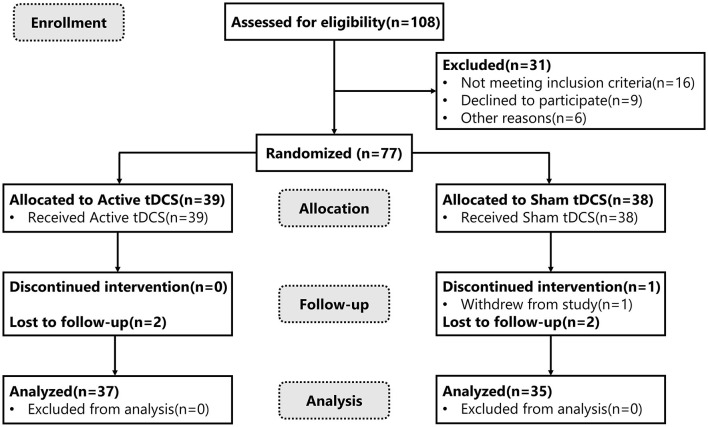
CONSORT diagram of study flow. tDCS, transcranial direct current stimulation.

A total of 108 stroke patients were assessed for eligibility, of whom 77 patients met the inclusion and exclusion criteria, and they were randomly assigned to either the tDCS group (*n* = 39) or the control group (*n* = 38). It is noteworthy that two patients in the tDCS group dropped out of the follow-up, while in the control group, one patient did not complete the study and two dropped out of the follow-up. In total, 72 patients completed the 4-week trial and follow-up (37 in the tDCS group and 35 in the control group) (Figure 2). There were no significant differences between the two groups in age, gender, stroke etiology, paralyzed side, duration of stroke event, stroke location, and baseline FMA-UE score (*P* > 0.05) (Table 1).

**Table 1:** Baseline characteristics of the patients.

	**tDCS group (*n* = 37)**	**Control group (*n* = 35)**	***P***
**Age, years**	56.3 (2.1)	56.8 (2.4)	0.87
**Age stage**			0.212
Young-adult (< 40 years)	4 (10.8%)	6 (17.1%)	
Middle-age (40–60 years)	18 (48.7%)	10 (28.6%)	
Old-age (>60 years)	15 (40.5%)	19 (54.3%)	
**Sex**			0.056
Male	26 (70.3%)	31 (88.6%)	
Female	11 (29.7%)	4 (11.4%)	
**Time since the stroke, days**	49.6 (6.4)	48.3 (6.5)	0.884
**Stroke etiology**			0.995
Hemorrhagic	19 (51.4%)	18 (51.4%)	
Ischemic	18 (48.6%)	17 (48.6%)	
**Stroke location**			0.916
Cortical	6 (16.2%)	6 (17.1%)	
Subcortical	31 (83.8%)	29 (82.9%)	
**Side of paresis**			0.611
Left	17 (45.9%)	14 (40%)	
Right	20 (54.1%)	21 (60%)	
**FMA-UE baseline score**	14.9 (1.9)	13.3 (2.5)	0.604
**Baseline impairment (FMA-UE score)**			0.419
Severe (0–28)	29 (78.4%)	30 (85.7%)	
Mild to moderate (29–66)	8 (21.6%)	5 (14.3%)	

Data are shown as *n* (%) or mean (SEM). tDCS, transcranial direct current stimulation; FMA-UE, Fugl-Meyer Assessment-Upper Extremity.

### Primary outcome

**Figure 3:**
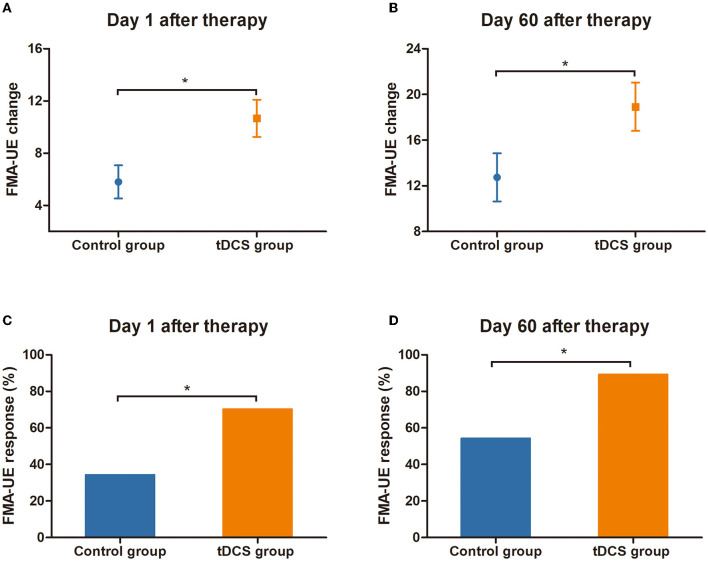
Response and change in FMA-UE score. **(A)** The change of FMA-UE score relative to the baseline at T1 (on the first day after the end of tDCS treatment). **(B)** The change of FMA-UE score relative to the baseline at T2 (at 60 days after the end of tDCS treatment). **(C)** FMA-UE response rate (change of ≥6 points from baseline) at T1. **(D)** FMA-UE response rate (change of ≥6 points from baseline) at T2. *Indicates a significant inter-group difference, *P* < 0.05. tDCS, transcranial direct current stimulation; FMA-UE, Fugl-Meyer Assessment-Upper Extremity.

At T0 (baseline, 1 day before tDCS treatment), the mean FMA-UE score in the tDCS group was 14.9 points (SEM = 1.9), and it was 13.3 points in the control group (SEM = 2.5). At T1 (the first day after 4 weeks of treatment), the mean FMA-UE score in the tDCS group was 25.6 points (SEM = 2.7), and it was 19.1 points in the control group (SEM = 3.0). At T2 (60 days after 4 weeks of treatment), the mean FMA-UE score in the tDCS group was 33.8 points (SEM = 3.0), and it was 26.0 points in the control group (SEM = 3.1). Regarding changes in the FMA-UE score from baseline to the first day after the therapy, the mean FMA-UE score increased by 10.7 points (SEM = 1.4) in the tDCS group and by 5.8 points (SEM = 1.3) in the control group. The FMA-UE score in the tDCS group was significantly higher than that in the control group (the difference between the two groups was 4.9 points, *P* = 0.013). Compared with the baseline, the mean FMA-UE score increased by 18.9 points (SEM = 2.1) in the tDCS group and by 12.7 points (SEM = 2.1) in the control group at 60 days after the completion of clinical treatment, which was also significantly higher than that in the control group (difference between the two groups was 6.2 points, *P* = 0.043) (Figure 3; Table 2).

**Table 2:** Comparison of FMA-UE score, FMA-UE changes, and FMA-UE response rates between the tDCS and control groups.

	**tDCS group (*n* = 37)**	**Control group (*n* = 35)**	***P***
**FMA-UE score**			
FMA-UE at T0	14.9 (1.9)	13.3 (2.5)	0.604
FMA-UE at T1	25.6 (2.7)	19.1 (3.0)	0.115
FMA-UE at T2	33.8 (3.0)	26.0 (3.1)	0.077
**Primary outcome**			
Change in FMA-UE at T1	10.7 (1.4)	5.8 (1.3)	**0.013**
Change in FMA-UE at T2	18.9 (2.1)	12.7 (2.1)	**0.043**
**Secondary outcome**			
FMA-UE response rate at T1	26 (70.3%)	12 (34.3%)	**0.002**
FMA-UE response rate at T2	33 (89.2%)	19 (54.3%)	**0.001**

Data are shown as *n* (%) or mean (SEM). tDCS, transcranial direct current stimulation. FMA-UE, Fugl-Meyer Assessment-Upper Extremity. T1 was the first day after tDCS treatment. T2 was 60 days after the end of tDCS treatment. FMA-UE response rate was defined as an increase in the FMA-UE score of 6 points or more. The bold values provided in the column of *P* value in the table represent significant differences (*P* < 0.05).

### Secondary outcome

Compared with the control group, a clinically meaningful response to the FMA-UE score on the first day after the end of tDCS therapy was 26/37 (70.3%) in the tDCS group and 12/35 (34.3%) in the control group, with a between-group difference of 36.0 % (*P* = 0.002). At 60 days after completion of the tDCS treatment, the clinically meaningful response of the FMA-UE score was 33/37 (89.2%) in the tDCS group and 19/35 (54.3%) in the control group; the difference between the two groups was 34.9% (*P* = 0.001) (Figure 3; Table 2).

All AEs during 4 weeks of tDCS treatment were recorded, including tingling, itching, headache, fatigue, nausea, and insomnia. There was no significant difference in the treatment-related AEs between the two groups (*P* > 0.05) (Table 3).

**Table 3:** Incidence of individual adverse events reported by the patients.

	**tDCS group (*n* = 37)**	**Control group (*n* = 35)**	***P***
Tingling sensation	8 (21.6%)	5 (14.3%)	0.419
Itching sensation	3 (8.1%)	4 (11.4%)	0.938
Headache	2 (5.4%)	0 (0%)	0.498
Fatigue	4 (10.8%)	2 (5.7%)	0.772
Nausea	0 (0%)	0 (0%)	/
Insomnia	0 (0%)	0 (0%)	/

Data are shown as *n* (%). tDCS, transcranial direct current stimulation.

### tDCS subgroup analysis of left/right paralyzes

To explore the effects of tDCS right cerebellar stimulation on functional recovery of different hemiplegic sides, 37 patients in the tDCS group were further divided into the right paralysis group (*n* = 20) and the left paralysis group (*n* = 17). There was no significant difference in the FMA-UE score between the two subgroups at baseline. Regarding changes in the FMA-UE score from baseline to the first day after the therapy, the mean FMA-UE score increased by 12.9 points (SEM = 2.0) in the right paralysis group and by 8.1 points (SEM = 1.9) in the left paralysis group (the difference between the two groups was 4.8 points, *P* = 0.098). Compared with the baseline, the mean FMA-UE score increased by 23.6 points (SEM = 3.1) in the right paralysis group and by 13.5 points (SEM = 2.3) in the left paralysis group at 60 days after completion of the tDCS treatment (the difference between the two groups was 10.1 points, *P* = 0.015) (Supplementary Table 1).

A clinically meaningful response to the FMA-UE score on the first day after the end of tDCS therapy was 17/20 (85.0%) in the right paralysis group and 9/17 (52.9%) in the left paralysis group, with a between-group difference of 32.1% (*P* = 0.032). At 60 days after completion of the tDCS treatment, the clinically meaningful response of the FMA-UE score was 19/20 (95.0%) in the right paralysis group and 14/17 (82.4%) in the left paralysis group, and the difference between the two groups was 12.6% (*P* = 0.211) (Supplementary Table 1).

### tDCS subgroup analysis of age

The efficacy of cerebellar tDCS in patients of different ages was also assessed using subgroup analysis. According to previous studies on stroke, someone aged less than 40 years was defined as a young adult, 40–60 years as middle-aged, and over 60 years as old-aged (19, 20). Due to the small number of young-adult patients in this study (*n* = 4), young-adult patients and middle-aged patients were combined into young-/middle-aged patients. Therefore, patients in the tDCS group were subdivided into a young-/middle-aged group (*n* = 22) and an old-aged group (*n* = 15), and there was no significant difference in the FMA-UE score between the two groups at baseline. Regarding changes in the FMA-UE score from baseline to the first day after the therapy, the mean FMA-UE score increased by 10.5 points (SEM = 2.1) in the young-/middle-aged group and by 10.9 points (SEM = 1.8) in the old-aged group (the difference between the two groups was 0.4 points, *P* = 0.884). Compared with the baseline, the mean FMA-UE score increased by 18.1 points (SEM = 2.8) in the young-/middle-aged group and by 20.1 points (SEM = 3.3) in the old-aged group at T2 (the difference between the two groups was 2.0 points, *P* = 0.660) (Supplementary Table 2).

A clinically meaningful response to the FMA-UE score on the first day after the end of tDCS therapy was 15/22 (68.2%) in the young-/middle-aged group and 11/15 (73.3%) in the old-aged group, with a between-group difference of 5.1% (*P* = 0.735). At 60 days after completion of the tDCS treatment, the clinically meaningful response of the FMA-UE score was 20/22 (90.9%) in the young-/middle-aged group and 13/15 (86.7%) in the old-aged group, and the difference between the two groups was 4.2% (*P* = 0.686) (Supplementary Table 2).

## Discussion

In this 3-month randomized, double-blind, sham-stimulation-controlled trial, it was found that cerebellar tDCS promoted recovery of upper limb motor function in stroke patients. Meanwhile, there was no significant difference in AEs between the two groups. To the best of our knowledge, this is the first RCT to explore the efficacy of cerebellar tDCS on upper limb motor dysfunction after stroke.

Currently, the treatment of motor function with tDCS in stroke patients concentrates on stimulating M1. Studies have shown that M1 tDCS stimulation could improve the motor function of the upper limbs in stroke patients (9, 21, 22). However, the motor function of the human body is not only regulated by M1 but also there are other functional cerebral regions involved in the regulation of movement. The cerebellum is one of the important motor regulation centers of the central nervous system (CNS), which is closely associated with the performance of skilled hand movement, limb coordination, gait, and cognitive function (23, 24). Researchers have paid more attention to the cerebellum as a promising stimulation target. Some studies have applied non-invasive brain stimulation techniques to the cerebellum and found that it could improve the balance function and postural stability of the elderly, patients with PD, and spinocerebellar ataxia (13, 14, 25). Yosephi et al. compared the efficacy of postural training with M1 or cerebellar tDCS and found that stimulation of the cerebellum could more significantly improve postural control or balance in older adults who were at a high risk of falling (14). It has also been demonstrated that cerebellar tDCS could enhance the retention of fine motor skills and improve the accuracy of motor skills (26, 27). Recently, there have been several studies on the application of cerebellar tDCS in stroke patients. Studies have found that cerebellar tDCS could improve the ability of picture naming in patients with post-stroke aphasia (28, 29). Zandvliet et al. first reported the short-term effect of cerebellar tDCS on balance in patients with chronic stroke and found that it could improve standing balance performance (30). The bipolar bilateral tDCS of the cerebellar dentate nucleus positively affects goal-directed weight shifting and postural control in stroke patients (31). Although cerebellar tDCS has a positive effect on the recovery of balance and posture control in stroke patients, there is no relevant study on the impact of cerebellar tDCS on upper limb motor dysfunction after stroke. In the present study, it was found, for the first time, that cerebellar tDCS could promote the recovery of upper limb motor dysfunction in stroke patients, which provided a new reference for the rehabilitation treatment of upper limb motor dysfunction after stroke.

To the best of our knowledge, no study has indicated whether the effect of cerebellar stimulation is superior to conventional M1 stimulation. In the present study, it was demonstrated that the FMA-UE score increased by 10.7 points at the end of 4 weeks of cerebellar tDCS treatment, and it increased by 18.9 points at 60 days after treatment. In a study that assessed the relationship between M1 tDCS and the recovery of upper limb motor function in stroke patients, the FMA-UE score increased by an average of approximately 10.1 points after 2 weeks of tDCS treatment (32). In another study, the FMA-UE score of patients with subacute stroke increased by 9.3 points after 3 weeks of M1 tDCS treatment (33). Although the treatment time in the present study was 1–2 weeks longer than that in the other two studies, the rehabilitation effect of cerebellar tDCS treatment on upper limb motor function in stroke patients may have a similar efficacy to that of conventional M1 stimulation. Future research is needed to confirm this finding.

The mechanism indicating how tDCS applied to the cerebellum could improve upper limb motor dysfunction after stroke has remained unclear. Motor learning is an adaptive behavioral change under the control of the CNS, which is crucial to the rehabilitation of motor function after a stroke (24, 34, 35). The cerebellum is one of the important components involved in motor learning. It may promote motor learning by predicting and accounting for systematic changes to the body or the environment, resulting in the correction of errors on a trial-by-trial basis (11, 12, 36). In our previous animal experiments, we found that the cerebellum could independently support simple eyeblink conditioning, which is of great significance for understanding the mechanism of the cerebellum in motor learning (10). The motor learning function of the cerebellum mainly involves Purkinje cells and granule cells (37). They may participate in motor learning by regulating signal transmission through long-term depression (LTD) and long-term potentiation (LTP). tDCS can produce LTD or LTP effects between synapses by delivery of weak and continuous direct current stimulation to the cerebellum, thereby changing synaptic plasticity. In behavioral research, the relationship between cerebellar tDCS and motor learning has been studied using various tasks. It has been found that tDCS acting on the cerebellum could regulate the acquisition of conditional eyeblink responses, improve the adaptation to the visuomotor transformation, and enhance locomotor adaptation (23, 38, 39). A recent study showed that age-related motor learning deficits could be diminished by cerebellar tDCS stimulation in older adults (40). It was revealed that cerebellar tDCS has the effect of regulating cerebellar motor learning, which can accelerate the acquisition of motor function and improve the accuracy of movement. This mode of regulation is closely associated with the polarity of cerebellar stimulation. Anodal tDCS is considered a technique to increase neuronal excitability, which can improve the ability of the cerebellum to learn from error and accelerates the learning process, while cathodal stimulation shortens the learning process of the cerebellum (38, 41). In the present study, it was found that anodal cerebellar tDCS promoted the recovery of upper limb motor function in stroke patients. Collectively, this effect could be attributed to the enhanced motor learning ability of the cerebellum by anodal tDCS.

In the present study, the right cerebellum was selected as the stimulation site of the cerebellum. This was based on previous studies that have shown that the right cerebellum was closely correlated with motor learning (23, 26, 27, 39, 41). However, there is no widely accepted standard for the selection of cerebellar electrode placement. The contra-lesional cerebellar hemisphere (30, 42), the ipsilesional cerebellar hemisphere (43), and the right cerebellum (28, 29) were all stimulatory targets selected by non-invasive brain stimulation techniques. It is not fully clear how to select the stimulation target for patients with different sides of paralysis. In the present study, patients receiving right cerebellar tDCS treatment were divided into two subgroups (left paralysis subgroup and right paralysis subgroup). The therapeutic effects were compared between the two subgroups. The results suggested that right cerebellar tDCS was more effective for right paralysis (left hemisphere stroke in the cerebrum) than left paralysis (right hemisphere stroke in the cerebrum). According to the aforementioned results, right cerebellar stimulation may be a better option for patients with left-hemisphere stroke in the cerebrum.

Age is one of the factors affecting the prognosis of stroke patients (44, 45). However, it is unclear whether age can affect the efficacy of non-invasive brain stimulation techniques. Kim et al. divided patients into a responded group and a non-responded group according to their responsiveness to rTMS treatment (46), and the results showed that the age in the responded group was significantly lower than that in the non-responded group. In another high-frequency rTMS study with stroke patients, Chang et al. found that age tended to influence clinically significant changes in the FMA-UE score, while no significant difference was identified (*P* = 0.104) (47). In the present study, subgroup analysis was conducted to indicate whether there would be differences in the efficacy of cerebellar tDCS in different age-based groups. The results showed similar therapeutic efficacy in young-/middle-aged or elderly stroke patients. The following reasons could explain why there was no statistically significant difference between the two groups: (1) The average FMA-UE score at baseline in the young-/middle-aged group was 12.0 points, while it was 19.1 points in the elderly group. The degree of upper limb dysfunction in the young-/middle-aged group was more serious than that in the elderly group. (2) Enrollment of a limited number of subjects in the two subgroups is noteworthy.

Transcranial direct current stimulation treatment is based on constant, low-intensity direct current to modulate the activity of cerebral cortical neurons. The time of tDCS treatment, the current intensity of stimulation, and the area of electrode pads are all important factors that affect the efficacy and safety of tDCS (48, 49). Studies have shown that applying a stimulation protocol at a current intensity of 1–2 mA for 20–30 min is safe (50). According to MRI examination of subjects, tDCS stimulation lasting for an hour does not induce brain edema or alterations of the blood–brain barrier or cerebral tissue (51). Studies have also confirmed that tDCS stimulation does not cause changes in serum neuron-specific enolase, as an indicator of neuronal injury (52, 53). Moreover, tDCS treatment does not directly induce the generation of action potentials; thus, there is no risk of seizures. In the present study, the most common AEs of tDCS treatment were mild tingling and itching under the electrodes. Other AEs included headache, fatigue, nausea, and insomnia. The aforementioned symptoms were all mild and short-lived. They disappeared after the stimulation stopped or within a few hours. No special treatment was required. These AEs were consistent with those reported previously, and there were no significant differences between the two groups. Therefore, tDCS exhibited as a safe therapy in the present study.

The present study has some limitations. First, only a few evaluation indicators were used for the assessment of upper limb motor function in stroke patients. Although the FMA-UE score is the most commonly used evaluation index to evaluate upper extremity motor function after a stroke, a variety of evaluation indicators can still be used to comprehensively investigate upper extremity motor function after strokes, such as the Wolf Motor Function Test. Second, the follow-up time was short. Efficacy was assessed on the first day and 60 days after the end of the 4-week tDCS treatment, and long-term efficacy beyond 60 days was not followed up. Third, due to the small sample size, no detailed study was conducted on different stroke sizes and durations.

## Conclusion

In conclusion, it was demonstrated that cerebellar anodal tDCS therapy could improve upper limb motor dysfunction in stroke patients with reasonable safety. In future studies, cerebellar tDCS should be used as an effective and safe supplementary therapy to promote the recovery of upper limb motor function in stroke patients.

## Data availability statement

The raw data supporting the conclusions of this article will be made available by the authors, without undue reservation.

## Ethics statement

The studies involving human participants were reviewed and approved by Ethics Committee of the Southwest Hospital of Army Medical University. The patients/participants provided their written informed consent to participate in this study.

## Author contributions

JH, PX, and HL conceived and designed the study. QG, XD, HC, and JH analyzed the experimental data and drafted the manuscript. RY, YC, JL, YZ, ZF, XW, and YZ collected the experimental data. QG, PX, JH, and JQ revised the manuscript for important intellectual content. All authors contributed to the article and approved the submitted version.
