# Supplementary material for: Effects of cerebellar transcranial direct current stimulation on rehabilitation of upper limb motor function after stroke
Source: Front Neurol. 2023 Mar 16;14:1044333. doi: 10.3389/fneur.2023.1044333 (PMC10060824; doi:10.3389/fneur.2023.1044333)
Supplement: Supplementary file 1 [file Table_1.DOCX]

Supplementary Material

# Supplementary Table:

**eTable 1.** Comparison of FMA-UE score, FMA-UE changes and FMA-UE response rates between left paralysis group and right paralysis group.

|  | Right paralysis group  **(n = 20)** | Left paralysis group  **(n = 17)** | ***P*** |
| --- | --- | --- | --- |
| **FMA-UE score** |  |  |  |
| FMA-UE at T0 | 15.1 (2.7) | 14.7 (2.8) | 0.931 |
| FMA-UE at T1 | 27.9 (3.6) | 22.8 (4.2) | 0.363 |
| FMA-UE at T2 | 38.6 (3.9) | 28.2 (4.4) | 0.085 |
| **Primary outcome** |  |  |  |
| Change in FMA-UE at T1 | 12.9(2.0) | 8.1(1.9) | 0.098 |
| Change in FMA-UE at T2 | 23.6(3.1) | 13.5(2.3) | **0.015** |
| **Secondary Outcome** |  |  |  |
| FMA-UE Response rate at T1 | 17 (85.0%) | 9 (52.9% ) | **0.032** |
| FMA-UE Response rate at T2 | 19 (95.0%) | 14 (82.4%) | 0.211 |

Data are shown as n (%) or mean (SEM). tDCS= transcranial direct current stimulation. FMA-UE= Fugl-Meyer Assessment-Upper Extremity. T1 was the first day after tDCS treatment. T2 was 60 days after the end of tDCS treatment. FMA-UE Response rate was define an increase in the FMA-UE score of 6 points or more.

**eTable 2.** Comparison of FMA-UE score, FMA-UE changes and FMA-UE response rates between young/middle-aged group and old-aged group.

|  | Young/middle-aged group **(n = 22)** | Old-aged group  **(n = 15)** | ***P*** |
| --- | --- | --- | --- |
| **FMA-UE score** |  |  |  |
| FMA-UE at T0 | 12.0 (2.1) | 19.1 (3.5) | 0.076 |
| FMA-UE at T1 | 22.5 (3.5) | 30.0 (4.3) | 0.185 |
| FMA-UE at T2 | 30.2 (3.8) | 39.1 (4.7) | 0.147 |
| **Primary outcome** |  |  |  |
| Change in FMA-UE at T1 | 10.5 (2.1) | 10.9 (1.8) | 0.884 |
| Change in FMA-UE at T2 | 18.1 (2.8) | 20.1 (3.3) | 0.660 |
| **Secondary Outcome** |  |  |  |
| FMA-UE Response rate at T1 | 15 (68.2%) | 11 (73.3%) | 0.735 |
| FMA-UE Response rate at T2 | 20 (90.9%) | 13 (86.7%) | 0.686 |

Data are shown as n (%) or mean (SEM). tDCS= transcranial direct current stimulation. FMA-UE= Fugl-Meyer Assessment-Upper Extremity. T1 was the first day after tDCS treatment. T2 was 60 days after the end of tDCS treatment. FMA-UE Response rate was define an increase in the FMA-UE score of 6 points or more.

**
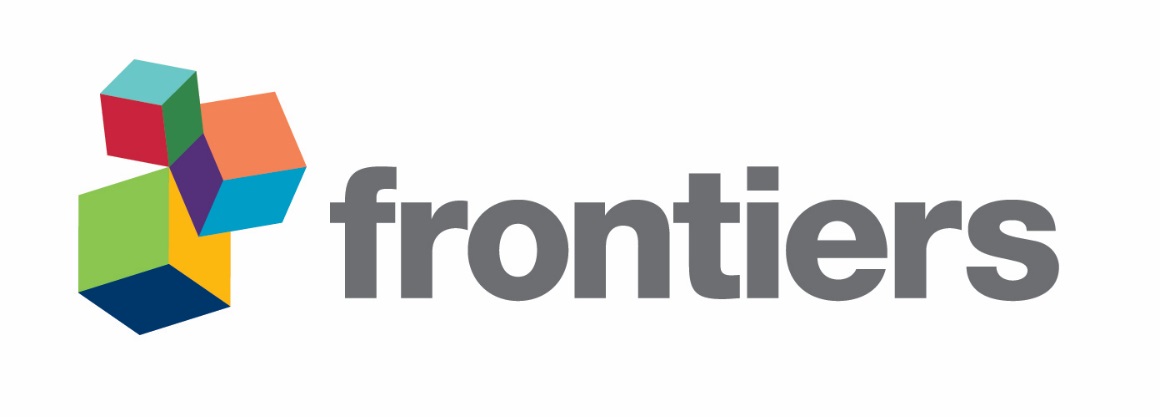
**
